# Impact of a multidimensional infection control approach on central line-associated bloodstream infections rates in adult intensive care units of 8 cities of Turkey: findings of the International Nosocomial Infection Control Consortium (INICC)

**DOI:** 10.1186/1476-0711-12-10

**Published:** 2013-05-04

**Authors:** Hakan Leblebicioglu, Recep Öztürk, Victor Daniel Rosenthal, Özay Arıkan Akan, Fatma Sirmatel, Davut Ozdemir, Cengiz Uzun, Huseyin Turgut, Gulden Ersoz, Iftíhar Koksal, Asu Özgültekin, Saban Esen, Fatma Ulger, Ahmet Dilek, Hava Yilmaz, Yalim Dikmen, Gökhan Aygún, Melek Tulunay, Mehmet Oral, Necmettin Ünal, Mustafa Cengiz, Leyla Yilmaz, Mehmet Faruk Geyik, Ahmet Şahin, Selvi Erdogan, Suzan Sacar, Hülya Sungurtekin, Doğaç Uğurcan, Ali Kaya, Necdet Kuyucu, Gürdal Yýlmaz, Selçuk Kaya, Hülya Ulusoy, Asuman İnan

**Affiliations:** 1 Ondokuz Mayis University Medical School, Samsun, Turkey; 2 Istanbul University Cerrahpasa Medical School, Istanbul, Turkey; 3 International Nosocomial Infection Control Consortium, Buenos Aires, Argentina; 4 Ankara University School of Medicine Ibni-Sina Hospital, Ankara, Turkey; 5 Faculty of Medicine, Harran University, Sanliurfa, Turkey; 6 Duzce University Medical School Infectious Diseases and Clinical Microbiology, Duzce, Turkey; 7 German Hospital, Istanbul, Turkey; 8 Pamukkale University, Denizli, Turkey; 9 Faculty of Medicine, Mersin University, Mersin, Turkey; 10 Karadeniz Technical University School of Medicine, Trabzon, Turkey; 11 Haydarpasa Numune Training and Research Hospital, Istanbul, Turkey

**Keywords:** Catheter related infections, Central line-associated bloodstream infection, Device-associated infections, Healthcare-associated infections, Bundle, International nosocomial infection control consortium, Multidimensional approach, Hand hygiene, Developing countries, Limited-resource countries

## Abstract

**Background:**

Central line-associated bloodstream infections (CLABs) have long been associated with excess lengths of stay, increased hospital costs and mortality attributable to them. Different studies from developed countries have shown that practice bundles reduce the incidence of CLAB in intensive care units. However, the impact of the bundle strategy has not been systematically analyzed in the adult intensive care unit (ICU) setting in developing countries, such as Turkey. The aim of this study is to analyze the impact of the International Nosocomial Infection Control Consortium (INICC) multidimensional infection control approach to reduce the rates of CLAB in 13 ICUs of 13 INICC member hospitals from 8 cities of Turkey.

**Methods:**

We conducted active, prospective surveillance before-after study to determine CLAB rates in a cohort of 4,017 adults hospitalized in ICUs. We applied the definitions of the CDC/NHSN and INICC surveillance methods. The study was divided into baseline and intervention periods. During baseline, active outcome surveillance of CLAB rates was performed. During intervention, the INICC multidimensional approach for CLAB reduction was implemented and included the following measures: 1- bundle of infection control interventions, 2- education, 3- outcome surveillance, 4- process surveillance, 5- feedback of CLAB rates, and 6- performance feedback on infection control practices. CLAB rates obtained in baseline were compared with CLAB rates obtained during intervention.

**Results:**

During baseline, 3,129 central line (CL) days were recorded, and during intervention, we recorded 23,463 CL-days. We used random effects Poisson regression to account for clustering of CLAB rates within hospital across time periods. The baseline CLAB rate was 22.7 per 1000 CL days, which was decreased during the intervention period to 12.0 CLABs per 1000 CL days (IRR 0.613; 95% CI 0.43 – 0.87; P 0.007). This amounted to a 39% reduction in the incidence rate of CLAB.

**Conclusions:**

The implementation of multidimensional infection control approach was associated with a significant reduction in the CLAB rates in adult ICUs of Turkey, and thus should be widely implemented.

## Background

The burden of central line-associated bloodstream infection (CLAB) in critically ill patients has been widely addressed in the scientific literature worldwide. According to studies from developed [1] and developing countries [2,3], including Turkey [4], the most serious clinical consequences attributable to CLAB are increased mortality rates [1], significant morbidity [1], and increased LOS [2,5,6]. From an economic perspective, CLABs are also responsible for significant increases in healthcare costs, as reported in both developed [1] and developing countries [2,5], but there are not available published data on costs of CLAB from Turkey.

The results reported from hospitals members of the International Nosocomial Infection Control Consortium (INCC) revealed that device-associated healthcare-acquired infections (DA-HAI) rates in the intensive care units (ICUs) of limited-resources countries are 3 to 5 times higher than rates in the ICUs of high-income countries [7-10]. However, most hospitals in limited-resource countries do not implement basic infection control programs, resulting in a general unawareness of the incidence of CLAB at their healthcare facilities [11].

In addition, the socio-economic level of a country and hospital type were reported to have an impact on DA-HAI rates in the ICU settings of developing countries in two studies [12,13]. As regards hospital type, CLAB rates in neonatal ICUs from public and academic hospitals were significantly higher than in private hospitals: 14.3 and 14.6 vs.10.8 CLABs per 1000 CL-days [12]. With regard to the country socioeconomic level, in a study conducted in pediatric ICUs it was shown that lower-middle-income countries had higher CLAB rates than upper middle-income countries (12.2 vs. 5.5 per 1000 CL-days) [13]. Similarly, CLAB rates in neonatal ICUs were shown to be higher in low-income countries than in lower-middle and upper-middle-income countries [12].

In the developed countries, it has been demonstrated that surveillance is fundamental to prevent CLABs, which can be reduced by more than 30% [14]. Implementing infection control bundles alone—including five interventions, such as 1- hand hygiene, 2- skin antisepsis with chlorhexidine, 3- maximal barriers, 4- insertion in subclavian vein, and 5 - timely central line (CL) removal—were associated with a reduction in the incidence density of CLAB in developed countries [15].

INICC supports hospitals in limited-resource countries in performing surveillance and reducing healthcare-associated infection rates. Hospitals from limited-resource countries contact INICC and then receive forms and manuals with guidance to implement effective surveillance and infection control programs. INICC also provides administrative and scientific support to upload, process, analyze and create charts and tables with the collected data.

With the aim of reducing these high CLAB rates, we implemented a multidimensional infection control program—which included specific interventions for CLAB prevention, such as a practice bundle, education, outcome surveillance, process surveillance, feedback of CLAB rates, as well as performance feedback of infection control practices—in 13 adult ICUs of 13 hospitals, in 8 cities of Turkey. The implementation of the INICC multidimensional program for CLAB prevention is based on the recommendations and guidelines published by the Society for Health Care Epidemiology of America (SHEA) and the Infectious Diseases Society of America (IDSA) in 2008 [16].

This study analyses the particular effect of this preventive multidimensional strategy on CLAB rates in the adult ICU setting of Turkey from September 2003 to January 2011.

## Methods

### Setting and study design

From September 2003 to January 2011, we conducted an active, prospective outcome and process surveillance before-after study in 13 Intensive Care Units (ICUs) in 13 hospitals members of the INICC in 8 cities of Turkey. Each hospital had been actively participating in the INICC Surveillance Program for at least 4 months. Every hospital had an infection control team (ICT) comprised of at least a medical doctor with formal education and background in internal medicine, critical care, infectious diseases, and/or hospital epidemiology, and infection control professionals (ICPs).

The study was divided in 2 periods: baseline period and intervention period.

The Institutional Review Board at each hospital approved the study protocol.

### Baseline period

The baseline period included only the performance of outcome surveillance and process surveillance. The length of the baseline period is three months due to the following three reasons:

1. This is the time needed to conduct the following activities at INICC headquarters (HQs) in Argentina on a monthly basis: receiving those case report forms (CRF) filled at all participating ICUs from Turkey; conducting a validation process of filled CRFs; sending queries to participating ICUs; receiving and analyzing replies to queries; uploading CRFs data with proprietary INICC software in Argentina; analyzing uploaded data; producing monthly reports containing charts and tables with the results of outcome and process surveillance; sending monthly reports to each ICUs; presenting the monthly report of outcome and process surveillance data to health care workers (HCWs) working at the participating ICUs in monthly infection control meetings, with the aim of providing feedback on CLAB rates and consequences and performance feedback and increase the awareness about CLABs to improve compliance with infection control practices.

2. Sample size of patients and number of months of data collection during baseline period is sufficient enough to compare with sample size of patients and number of months of data collection during intervention period. From a statistical perspective, the issue is addressed by considering the changes in rates over time. The relatively short baseline period may impact the standard error of our estimates. But we found that this will not cause a bias in the results, because there will not be systematic differences between the two groups.

3. Our priority is to start intervention as early as possible in order to achieve the desired results, such as CLAB rate and mortality rate reduction, as soon as possible.

### Intervention period

The intervention period was initiated after three months of participation in the INICC outcome and process surveillance program. This is a cohort study, and for that reason, each ICU joined INICC program at different moments. Thus, by the time we analyzed the impact of the INICC intervention, we had ICUs with different lengths of intervention periods. The average length of the intervention period was 15.6 months ± SD 9.2 (range 4 – 36).

### INICC multidimensional infection control approach

The INICC multidimensional infection control approach includes the following: 1- bundle of infection control interventions, 2- education, 3- outcome surveillance, 4- process surveillance, 5- feedback of CLAB rates, and 6- performance feedback of infection control practices.

### Components of central line-care bundle for CLAB

1. Perform hand hygiene before CL insertion or manipulation [17].

2. Use sterile gauze or transparent sterile dressing to cover insertion site [16].

3. Maintain good condition of sterile dressing. Change gauze every 48 hours and transparent dressing every 7 days [16].

4. Remove CL as early as possible, when not necessary [16].

5. Change administration set every 96 hours; unless used for fat, nutrition or blood precuts, and in this cases changed every 24 hours [16].

6. Use a chlorhexidine-based antiseptic for skin preparation [16].

7. Preferably use subclavian vein [16].

8. Use an all-inclusive catheter cart or kit [18].

9. Use maximal sterile barrier precautions during CL insertion [16].

10. Avoid using several times those vials meant to be use only once [16].

11. Disinfect line hubs, needleless connectors, and infection ports before accessing the CL [16].

Some other effective interventions were discussed, but not applied because of budget limitations; namely, the following five (5) practices were partially applied or not applied:

1. Use of split septum instead of mechanical valves or three ways stopcock [16].

2. Use of chlorhexidine impregnated sponge at insertion site [16].

3. Daily bath with chlorhexidine [16].

4. Use of antimicrobial impregnated catheters [16].

5. Use of closed collapsible flexible containers instead of open semi-rigid vented or glass vented IV containers [19].

### Education

Monthly sessions of education provided by ICP to the HCWs in charge of the insertion, care, and maintenance of CLs for CLAB prevention based on SHEA and IDSA guidelines to prevent CLAB [16].

### INICC surveillance methods

The INICC Surveillance Program includes two components: outcome surveillance (DA-HAI rates and their adverse effects, including mortality rates) and process surveillance (adherence to hand hygiene and other basic preventive infection control practices) [20].

Investigators were required to complete outcome and process surveillance forms at their hospitals, which were then sent to the INICC headquarters office in Buenos Aires, for their monthly analysis.

### Outcome surveillance

Outcome Surveillance included rates of CLAB per 1000 CL-days, use of invasive devices (CL, mechanical ventilator, and urinary catheter), severity illness score, underlying diseases, use of antibiotics, culture taken, microorganism profile, bacterial resistance, length of stay, mortality in their ICUs [20].

CLAB definitions and surveillance methods were performed applying the definitions for healthcare-associated infection (HAI) developed by the U.S. Centers for Disease Control and Prevention (CDC) for the National Healthcare Safety Network (NHSN) program [21]. In limited-resource countries, the usual practice is taking blood cultures after starting antibiotic treatment, or not taking blood cultures at all. This results in having patients with CLAB but without laboratory evidence of CLAB. For that reason, we decided to continue using “clinical sepsis” as well as “laboratory confirmed bloodstream infections” as definition criteria for “CLAB”, as historically used by the CDC NNIS [22].

Additionally, INICC methods were adapted to the limited-resource setting of developing countries, due to their different socioeconomic status [20]. ASIS score was used instead of APACHE II score due to budget limitations of participating ICUs from this limited-resource country. Thus, we decided to use ASIS score, as historically used by the CDC NNIS [22].

### Definitions

#### Laboratory confirmed CLAB

When CLAB is suspected, the CL is removed aseptically and the distal 5 cm of the catheter is amputated and cultured, using the standardized semi quantitative method [21,22]. Concomitant blood cultures are drawn percutaneously in most cases. In each hospital, standard laboratory methods are used to identify microorganisms, and standardized susceptibility testing is performed [21,22]. A patient with a CL who has a recognized pathogen isolated from one or more percutaneous blood cultures after 48 hours of catheterization; the pathogen cultured from the blood is not related to an infection at another site; and patient has one or more of the following signs or symptoms: fever (≥38°C), chills, or hypotension. With skin commensals (diphtheroids, *Bacillus* spp., *Propionibacterium* spp., coagulase-negative staphylococci or micrococci), the organism has been recovered from two or more separate blood cultures [21-23].

#### Clinically suspected CL-associated bloodstream infection

A patient with a CL who has at least one of the following clinical signs, with no other recognized cause: fever (≥38°C), hypotension (systolic blood pressure ≤90 mmHg) or oliguria (≤20 mL/hr), but blood cultures were either not obtained or no organisms were recovered from blood cultures; there is no apparent infection at another site; and the physician institutes antimicrobial therapy [21,22].

### Process surveillance

Process surveillance was designed to assess compliance with easily measurable key infection control practices, such as surveillance of compliance rates for hand hygiene practices and specific measures for the prevention of CLAB [20].

Because of budget limitations, only five out of eleven components of the bundle were monitored:

1. Hand hygiene (HH) compliance rate was based on the frequency with which HH was performed as indicated in HCWs infection control training. Observing ICPs were trained to record HH opportunities and compliance on a form, during randomly selected observation periods of 30 minutes to 1 hour, 3 times a week. In particular, the INICC direct observation comprised the “Five Moments for Hand Hygiene” as recommended by the World Health Organization (WHO). The “Five Moments” included the monitoring of the following moments: (1) before patient contact, (2) before an aseptic task, (3) after body fluid exposure risk, (4) after patient contact, and (5) after contact with patient surroundings [24]. Although HCWs knew that hand hygiene practices were regularly monitored, they were not informed of the schedule for HH observations.

2. Data on compliance with CL-care measures were recorded 5 days a week on a form that evaluated if infection control procedures were correctly carried out by the HCW. The ICP observing the activity in the AICU filled a standardized form that contained the following data: total number of inserted CLs for each patient for the whole ICU; total number of dressing placed to protect the puncture site in order to evaluate number of patients with sterile dressing [20].

3. Also the ICP filled a standardized form that contained total number of dressings in correct condition, evaluating if the dressing was clean, dry and correctly adhered to the puncture site, so as to evaluate number of dressing in correct condition [20].

4. Also the ICP filled a standardized form that contained total number of cases in which the dates in the administration set were written, with the aim of measuring the number of patients with number of days of the administration set in place, and evaluating if the set was replaced by or before 96 hours [20].

5. Finally, the ICP filled a standardized form including the date of insertion and removal of each CL, to evaluate number of days of CL inserted, and the earliest possible removal CL when not necessary.

### Feedback of DA-HAI rates

Upon processing the hospitals’ outcome surveillance data on a monthly basis, the INICC Research Team, at INICC Headquarters located in Buenos Aires, prepares and sends to each ICT a final report on the results of outcome surveillance rates; that is, monthly DA-HAI rates, length of stay, bacterial profile and resistance, and mortality [20].

Feedback of DA-HAI rates is provided to HCWs working in the AICU by communicating the outcomes of the patients. The resulting rates are reviewed by the ICT at monthly meetings, where charts are analyzed, and statistical graphs and visuals are posted inside the ICU, to provide an overview of rates of DA-HAIs. This infection control tool is key to increase awareness about outcomes of patients at their ICU, enable the ICT and ICU staff to focus on the necessary issues and apply specific strategies for improvement of high DA-HAI rates.

### Performance feedback

Upon processing the hospitals’ process surveillance data on a monthly basis, the INICC Research Team, at INICC Headquarters located in Buenos Aires, prepares and sends to each ICT a final report on the results of process surveillance rates, including compliance with hand hygiene, and care of CL [20].

Performance feedback is provided to HCWs working in the AICU by communicating the assessment of practices routinely performed by them. The resulting rates are reviewed by the ICT at monthly meetings, where charts are analyzed, and statistical graphs and visuals are posted inside the ICU, to provide an overview of rates measuring compliance with infection control practices. This infection control tool is key to enable the ICT and ICU staff to focus on the necessary strategies for improvement of low compliance rates.

### Statistical methods

Patients’ characteristics during baseline and during intervention period in each ICU were compared using Fisher’s exact test for dichotomous variables and unmatched Student’s *t*-test for continuous variables. Confidence intervals (CI) of 95% were calculated using VCStat (Castiglia). Relative risk (RR) ratios with 95% confidence intervals (CI) were calculated for comparisons of rates of CLAB using EPI Info V6. P-values <0.05 by two-sided tests were considered significant. We conducted two types of analysis to evaluate the impact of our intervention on CLAB rates. First, we performed an analysis to compare the data of the first three months (baseline period) with the remaining pooled months (intervention period), using RR, 95% CI and P value. Second, in order to analyze progressive CLAB rate reduction, we used Poisson regression. We divided the data into the first three months (baseline period), followed by a nine-month period (intervention period), and two annual follow-up periods for the second and third years. We compared the CLAB rates for each follow-up period with the baseline CLAB rate. For this comparison, we used as baseline data only those hospitals that contributed to follow-up in that period (i.e. excluding from the baseline hospitals with long lengths of follow-up that only contributed a shorter length of surveillance). We used random effects Poisson regression to account for clustering of CLAB rates within hospitals across time periods. These models were estimated using Stata 11.0. For this analysis we used IRR, 95% CI, and P value.

### Consent

Written informed consent was obtained from the patient for publication of this report and any accompanying images. Every hospital’s institutional review board agreed to the study protocol, and patient confidentiality was protected by codifying the recorded information, making it only identifiable to the ICT.

## Results

Over the whole study period, 4017 adult patients hospitalized for 42,749 days in 13 ICUs, from 13 hospitals, from 8 cities were enrolled, and 26,592 CL-days were collected. The participating hospitals were summarized and classified according to number of ICUs and type of hospital, and ICU characteristics by hospital. The first ICUs to participate were enrolled in September 2003, and the most updated data included our analysis dates from January 2011 (Tables 1 and 2).

**Table 1:** Characteristics of the participating intensive care units, and hospitals (from September 2003 to January 2011)

**Data**	**ICUs, n**	**ICU Patients, n**
**Type of ICU, n (%)**		
Cardiac Surgical	1 (8%)	172
Surgical	1 (8%)	222
Medical	2 (15%)	452
Adult Stepdown	2 (15%)	828
Medical Surgical	7 (54%)	2,343
All ICUs	13 (100%)	4,017
**Type of hospital, n (%)**		
Private Community	12 (92%)	3,996
Academic Teaching	1 (8%)	21
All hospitals	13 (100%)	4,017

ICU, Intensive Care Unit.

**Table 2:** Characteristics of the participating intensive care units by hospital (from September 2003 to January 2011)

**Hospital**	**Type of ICU**	**Bed-days, n**	**ICU Beds, n**
Hospital 1	Medical	1114	8
Hospital 2	Medical Surgical	353	11
Hospital 3	Medical Surgical	2136	15
Hospital 4	Medical Surgical	7630	24
Hospital 5	Adult Stepdown	6532	16
Hospital 6	Medical Surgical	393	16
Hospital 7	Medical Surgical	7229	8
Hospital 8	Cardiac Surgical	801	11
Hospital 8	Medical	2234	14
Hospital 8	Adult Stepdown	3510	14
Hospital 8	Surgical	1208	11
Hospital 9	Medical Surgical	6113	8
Hospital 10	Medical Surgical	3496	14

ICU, Intensive Care Unit.

Patients’ characteristics, such as age, gender, abdominal surgery, cardiac surgery, trauma, previous infections, endocrine diseases, chronic obstructive pulmonary diseases, renal impairment, hepatic failure, thoracic surgery, and stroke were similar during both periods. CL duration, CL use, and ASIS mean score were higher during the intervention period. This means patients had higher intrinsic infection risk at the intervention period (Table 3).

**Table 3:** Characteristics of patients, hand hygiene compliance, central line care compliance, central line usage, central line-associated blood stream infection rates, in the baseline period and intervention period

**Patients’ characteristics**	**Baseline**	**Intervention**	RR*	**95% CI**	**P- Value**
Study period by hospital in months, mean ± SD (range)	3	15.6 ± 9.2 (4 – 36)			
Number of Patients	560	3457			
*Bed days, n	5517	37232			
**No. of CL days, n	3129	23463			
***CL use, mean	0.57	0.63	1.1	1.07 – 1.15	0.0001
CL duration, mean ± SD	5.6 ± 9.0	6.8 ± 11.0	-	-	0.014
Age, mean ± SD	54.1 ± 22.0	52.3 ± 21.4	-	-	0.08
ASIS score, mean ± SD	3.2 ± 1.1	3.6 ± 1.14	-	-	0.0001
Male	305 (57%)	2,048 (60%)	1.05	0.91 – 1.21	0.5
Female	230 (43%)	1379 (40%)	-	-	-
Abdominal Surgery, n (%)	58 (10%)	349 (10%)	0.97	0.74 – 1.3	0.84
Cardiac Surgery, n (%)	11 (2%)	72 (2%)	1.1	0.56 – 2.22	0.9
Trauma, n (%)	58 (10%)	357 (10%)	0.99	0.75 – 1.34	0.97
Previous Infections, n (%)	81 (14%)	415 (12%)	0.83	0.653 – 1.1	0.13
Endocrine diseases, n (%)	43 (8%)	250 (7%)	0.94	0.68 – 1.33	0.7
Chronic Obstructive, n (%)	150 (27%)	904 (26%)	0.98	0.82 – 1.17	0.8
Renal Impairment, n (%)	33 (6%)	180 (5%)	0.9	0.61 – 1.3	0.5
Hepatic Failure, n (%)	13 (2%)	50 (1%)	0.62	0.33 – 1.25	0.14
Thoracic Surgery, n (%)	27 (5%)	151 (4%)	0.91	0.6 – 1.42	0.62
Stroke, n (%)	14 (3%)	64 (2%)	0.74	0.4 – 1.43	0.3
Hand Hygiene compliance % (n/n)	32% (427/1328)	49% (5260/10786)	1.52	1.4 – 1.7	0.0001
Date on administration set % (n/n)	33% (1544/4656)	39% (14159/36472)	1.17	1.11 – 1.2	0.0001
Placed sterile dressing % (n/n)	78% (3617/4656)	90% (32895/36472)	1.2	1.12 – 1.2	0.0001
Correct condition of dressing % (n/n)	76% (3537/4656)	73% (26699/36472)	0.96	0.93 – 0.99	0.04
No. of CLAB, n	71	372			
CLAB Rate per 1000 CL days	22.7	15.85	0.7	0.54 – 0.91	0.008

SD, standard deviation; CL, central line; CLAB, Central line associated bloodstream infection; RR, relative risk; CI, confidence interval; ASIS, average severity of illness score.

*Bed-days are the total number of days that patients are in the ICU during the selected time period.

**CL-days: the total number of days of exposure to central line by all of the patients in the selected population during the selected time period.

***CL use ratios were calculated by dividing the total number of CL-days by the total number of bed-days.

In relation to compliance rates, during the intervention period, HH compliance improved significantly, as well as compliance with other measures, including presence of date on administration set, placed dressing, and condition of sterile dressing (Table 3).

During the baseline period, we recorded 3,129 CL-days, for a CL use mean of 0.57. There were 71 CLABs, for an overall baseline rate of CLAB of 22.7 CLABs per 1000 CL-days (Table 3).

Merging all data of the intervention period, during the implementation of the multidimensional infection control approach, we recorded 23,463 CL-days, for a CL use mean of 0.63, and there were 372 CLABs, for an incidence density of 15.85 CLABs per 1000 CL-days. These results showed a CLAB rate reduction from baseline by 30% (from 22.7 to 15.85 CLABs per 1000 CL-days; RR 0.70, 95% CI 0.54 – 0.91, P 0.008) (Table 3).

On the other hand, using Poisson regression, we found a progressive reduction in the rate of CLAB. The baseline CLAB rate (during the first three months of study) was progressively reduced during the intervention period to 12.3 CLABs per 1000 CL days, accounting for a 43% CLAB rate reduction (IRR 0.57; 95% CI 0.41 – 0.80; P 0.001) (Table 4).

**Table 4:** Central line associated blood stream infection rates stratified by the length of time that each intensive care unit has participated in the International Nosocomial Infection Control Consortium

**Time since joining INICC**	**ICUs, n**	**Central line days, n**	**CLAB, n**	**Crude CLAB rate/1000 CL days**	**IRR accounting for clustering by ICU**	**P value**
1-3 months (baseline)	13	3,129	71	22.7	1	-
4-12 months	13	9,751	170	17.4	0.79 (0.59 – 1.04)	0.103
Second year	11	7,287	123	16.9	0.63 (0.46 – 0.87)	0.004
Third year	6	6,425	79	12.3	0.53 (0.38 – 0.76)	0.001

Poisson regression.

INICC, International Nosocomial Infection Control Consortium; ICU, intensive care unit; CL, central line; CLAB, Central line associated bloodstream infection; IRR, incidence-rate ratio.

The microorganisms profile is shown in Table 5. The predominant microorganisms in both periods were *Staphylococcus aureus*, coagulase negative Staphylococci spp. and *Acinetobacter* spp.

**Table 5:** Microorganism related to central line associated blood stream infection in adult intensive care units in phase 1 (baseline period) and phase 2 (intervention period)

**Isolated Microorganisms**	**Baseline**	**Intervention**	**P.value**
*Acinetobacter* spp. % (n)	*14*.*5% (9)*	*23.2% (79)*	*0.1293*
*Candida* spp. % (n)	*9.7% (6)*	*8.2% (28)*	*0.7023*
*Citrobacter* spp. % (n)	*0.0% (0)*	*0.3% (1)*	-
*Corynobacter %* (n)	*0.0% (0)*	*0.6% (2)*	-
*E. Coli* spp. % (n)	*6.5% (4)*	*6.2% (21)*	*0.8429*
*Enterobacter* spp. % (n)	*6.5% (4)*	*3.5% (12)*	*0.4627*
*Enterococcus* spp. % (n)	*3.2% (2)*	*6.7% (23)*	*0.441*
*Haemophilius, spp.*	*0.0% (0)*	*0.6% (2)*	-
*Klebsiella* spp. % (n)	*3.2% (2)*	*6.2% (21)*	*0.5365*
*Proteus* spp. % (n)	*0.0% (0)*	*0.3% (1)*	-
*Pseudomonas* spp. % (n)	*8.1% (5)*	*10.9% (37)*	*0.5089*
*Staphylococcus aureus* spp. % (n)	*21.0% (13)*	*17.0% (58)*	*0.4516*
Coagulase-negative staphylococci spp. % (n)	*27.4% (17)*	*15.0% (51)*	*0.0159*
*Serratia* spp. % (n)	*0.0% (0)*	*0.6% (2)*	-
*Stenotrophomonas %* (n)	*0.0% (0)*	*0.3% (1)*	-
*Streptococcus %* (n)	*0.0% (0)*	*0.6% (2)*	-
**Total**	100% (62)	100% (341)	-

## Discussion

If compared with the rates of developed countries, the baseline rate of CLAB found in this study (22.7 per 1000 CL-days) was more than ten-fold higher than the US 1.1 CLAB rate per 1000 CL-days determined by the CDC/NSHN [25]; and more than ten-fold higher than the 1.4 CLAB rate determined by KISS [26].

In comparison with global CLAB rates from developing countries, our CLAB baseline rate was considerably higher than the fourth international INICC reports published in 2012 (6.8 CLABs per 1000 CL-days) [10]. Likewise, within the scope of other studies addressing the burden of CLABs in Turkey, our CLAB rate of our study was higher than the rate found in other two studies conducted in Turkey showing 17.6 CLABs per 1000 CL days [4], and 11.8 CLABs per 1000 CL days [27].

In studies performed by INICC member hospitals, it was shown that the implementation of a multidimensional approach for CLAB--which includes a bundle of interventions, education, outcome and process surveillance, feedback of CLAB rates, and performance feedback--resulted in significant reductions in rates of CLAB in Argentina (46.63 vs. 11.10 CLABs per 1000 CL-days) [28]; in Mexico (46.3 vs. 19.5 CLABs per 1000 CL-days) [29]; in adult ICUs (14.5 vs. 9.7 CLABs per 1000 CL-days) [30]; and in pediatric ICUs (10.7 vs. 5.2 CLABs per 1000 CL-days) [31].

The INICC multidimensional approach for CLAB included the following elements. First, the implementation of an infection prevention bundle based on the guidelines published by the SHEA and IDSA [16], which provide evidence-based recommendations and cost-effective infection control measures, which can be feasibly adapted to the ICU setting in developing countries. Second, education of HCWs about infection preventive measures. Third, CLAB outcome surveillance by applying the definitions for CLAB developed by the U.S. CDC/NHSN [21,22]. Fourth, CLAB process surveillance to monitor compliance with easily measurable infection control measures, including HH performance. Fifth, feedback of CLAB rates. Sixth, performance feedback of process surveillance, particularly, by reviewing and discussing charts results at monthly infection control meetings.

In our study, patients’ characteristics, such as age, gender, and underlying diseases showed similar patient intrinsic risk in both study periods. But ASIS score, CL use, and CL duration were higher during the intervention period, meaning that the patient intrinsic risks were higher in the intervention period. During the implementation of the INICC multidimensional approach, we found an improvement in process surveillance rates, with HH compliance improved by 52%, compliance with date on administration set improved by 17%, compliance with placed sterile dressing improved by 20%, and compliance with correct condition of dressing was high during both periods. During the study period, the high CLAB rate at baseline was reduced from 22.7 to 12.00 per 1000 CL-days, showing a 39% CLAB rate reduction and evidencing the effectiveness of the applied multidimensional approach.

Our study can be compared with an earlier bundle study [15], and a number of important differences between them can be mentioned. First, this previously published bundle included five elements. In contrast, we included eleven. Second, compliance was not measured for any of the bundle components, whereas we checked compliance of 5 bundle components. Third, characteristics of patients during baseline and intervention periods were not collected nor analyzed so as to check and compare such individual features, whereas we did and could find that our patients were statistically similar during both periods. Fourth, the follow-up period was 18 months, whereas we included a 36-month follow-up period. Fifth, intervention included only a bundle and a check list, whereas our study included the above-mentioned 6 simultaneous interventions. Finally, microorganisms responsible for CLAB were not provided, whereas in our study we included the CLAB microorganism profile for both baseline and intervention periods. The most important differences were measurements of the population’s features and compliance with bundle elements, which allowed us to analyze the real impact of our intervention by excluding confounders associated with patients’ characteristics and infection control practices.

Regarding the microorganisms profile, we identified a predominance *Staphylococcus aureus*, coagulase-negative staphylococci spp. and *Acinetobacter* spp. during both periods, which is similar to the findings of other studies conducted in limited-resource countries [7-10].

This study has several limitations. First, our findings cannot be generalized to all ICU patients from Turkey. However, this study proved that a multidimensional approach is fundamental to understand and fight against the adverse effects of CLAB in the ICU setting of Turkey. Second, the setting of three-month baseline period may be short and might have overestimated the effect of the intervention; however, during baseline period the sample size was good enough, and the confidence intervals for the baseline rate were narrow. Finally, because we did not count on the necessary resources, we were not able to differentiate between early and late onset infections; we could not quantify in detail all the interventions included in our multidimensional approach, such as education; and we could not quantify compliance with some of the components of our bundle. Therefore, we could not evaluate the components’ individual implications or other contextual factors related to the ICU or hospitals individually. Nevertheless, our main goal was to reduce the high baseline CLAB rates found in our ICU, and although our interventions were inexpensive, the individual evaluation would have required more allocation of time, contributing to unnecessary harm for ICU patients. Fortunately, as from January 2012, we have been able to collect all these process surveillance data.

## Conclusions

This is the first multicenter study to report a substantial reduction in CLAB rates in the ICU setting of Turkey, proving this kind of infection control approach successful. Despite higher patient intrinsic risk characteristics during the intervention period, a multidimensional approach including improved compliance with preventive measures for CLAB resulted in significant reductions in the CLAB incidence rate. Nevertheless, it is worth highlighting that the reduction in CLAB rates does not derive from surveillance itself. These systematically collected data should serve to guide ICPs in their strategies for improvement of patient care practices, such as performance feedback, as demonstrated in several previous studies conducted in limited resources countries [29,30,32,33].

We expect that these preventive strategies proven effective in the INICC AICUs of Turkey by means of the implementation of the multidimensional approach for CLAB prevention results in a wider acceptance of infection control programs in hospitals worldwide, leading to significant CLAB reductions. Through the INICC network, investigators are freely furnished with training and methodological tools to perform outcome and process surveillance, and to implement an effective infection prevention model for CLABs, and at the same time, the publication of these findings serves to foster relevant scientific evidence-based literature. For this reason, every hospital is invited to participate in the INICC project, which was set up to respond to the compelling need in the developing world to significantly prevent, control and reduce CLABs and their adverse effects.

## Competing interests

The authors declare that they have no competing interests related to this article. Every hospital’s Institutional Review Board agreed to the study protocol, and patient confidentiality was protected by codifying the recorded information, making it only identifiable to the infection control team.

## Authors’ contributions

Idea, conception and design: VDR Software development: VDR Assembly of data: VDR Analysis and interpretation of the data: VDR Epidemiological analysis: VDR Statistical analysis: VDR Administrative, technical, and logistic support: VDR Drafting of the article: VDR Critical revision of the article for important intellectual content: All authors. Final approval of the article: All authors. Provision of study patients: All authors. Collection of data: All authors. Funding: VDR and the Foundation to Fight against Nosocomial Infections funds all the activities at INICC head quarters.
